# Loss of Polo ameliorates APP-induced Alzheimer’s disease-like symptoms in *Drosophila*

**DOI:** 10.1038/srep16816

**Published:** 2015-11-24

**Authors:** Fei Peng, Yu Zhao, Xirui Huang, Changyan Chen, Lili Sun, Luming Zhuang, Lei Xue

**Affiliations:** 1 Institute of Intervention Vessel, Shanghai 10th People’s Hospital, Shanghai Key Laboratory of Signaling and Disease Research, School of Life Science and Technology, Tongji University, 1239 Siping Road, Shanghai 200092, China; 2 School of Life Science and Technology, Tongji University, 1239 Siping Road, Shanghai 200092, P.R. China

## Abstract

The amyloid precursor protein (APP) has been implicated in the pathogenesis of Alzheimer’s disease (AD). Despite extensive studies, little is known about the regulation of APP’s functions *in vivo*. Here we report that expression of human APP in *Drosophila*, in the same temporal-spatial pattern as its homolog APPL, induced morphological defects in wings and larval NMJ, larva and adult locomotion dysfunctions, male choice disorder and lifespan shortening. To identify additional genes that modulate APP functions, we performed a genetic screen and found that loss of Polo, a key regulator of cell cycle, partially suppressed APP-induced morphological and behavioral defects in larval and adult stages. Finally, we showed that eye-specific expression of APP induced retina degeneration and cell cycle re-entry, both phenotypes were mildly ameliorated by loss of Polo. These results suggest Polo is an important *in vivo* regulator of the pathological functions of APP, and provide insight into the role of cell cycle re-entry in AD pathogenesis.

Alzheimer’s disease (AD) is the most common neurodegenerative disease characterized by progressive and irreversible decline of memory, cognitive function and language skills, eventually culminating in dementia^1^,^2^. The neuro-pathological hallmarks of AD are neurofibrillary tangles (NFTs) containing hyperphosphorylated aggregates of the microtubule-associated protein Tau, and extracellular senile plaques (SPs) containing β-amyloid (Aβ) peptides^3^,^4^. The various species of Aβ peptides are derived from the amyloid precursor protein (APP), a large single-pass transmembrane protein expressed at high level in the brain^5^. APP is proteolytically processed in a highly complex fashion by a series of sequential proteases to generate a series of fragments, including Aβ peptides, N-terminal fragment (N-APP) and C-terminal intracellular domain (AICD)^5^,^6^. Mutations in APP have been associated with familial susceptibility to AD^7^,^8^, and augmented APP expression levels also increase the risk of AD^9^. In humans, the APP gene is located on chromosome 21. Interestingly, patients with Trisomy 21 (Down’s Syndrome) invariably develop AD^10^. Duplications of APP locus have also been discovered in some younger patients developing AD^11^. Though APP has been clearly implicated in the pathogenesis of AD, the genes that modulate APP’s functions remain largely unknown.

In the past decades, increasing evidence suggests that cell cycle re-entry plays a fundamental role in the pathogenesis of AD^12^,^13^,^14^. Neurons in the adult central neuronal system have classically been recognized as terminally differentiated, that are arrested in the G0 phase of cell cycle in a non-dividing and non-replicating state. However, in AD brain, vulnerable neurons display an activated cell cycle phenotype - abnormal elevation of cell cycle markers and re-expression of cell cycle regulators, suggesting these neurons have re-entered the cell cycle, yet they lack the ability to complete the cell cycle, which results in aberrant neuronal death^15^,^16^,^17^,^18^.

Polo kinases belong to a highly conserved Ser/Thr kinase family originally identified in *Drosophila melanogaster*^19^. Polo kinases contain the canonical serine/threonine kinase domain and a unique Polo Box Domain (PBD) that docks the kinases to target proteins^20^. Budding yeast and *Drosophila* each encodes only one Polo kinase, named as Cdc5 and Polo, respectively^21^. Five Polo-like kinases, Plk1, Plk2/SNK, Plk3/CNK/FNK, Plk4/SAK and Plk5, have been identified in mammals^22^. Polo kinases have been previously reported as key regulators of multiple aspects of the cell cycle, including mitotic entry, centrosome organization, spindle formation, chromosome segregation, mitotic exit and cytokinesis^23^,^24^,^25^,^26^,^27^,^28^,^29^,^30^,^31^. Intriguingly, Plk1 level is elevated in susceptible hippocampal and cortical neurons of AD patients^32^,^33^, yet the role of Plk1 in AD pathogenesis remains elusive.

*Drosophila* has been used as a powerful model organism to study the pathology and treatment of AD^34^,^35^,^36^,^37^,^38^. In the present study, we expressed human APP in the same pattern as its *Drosophila* homolog APPL, and observed morphological abnormalities in the adult wing and larval neuromuscular junction (NMJ), locomotion defect, shortened lifespan and impaired choice ability. These phenotypes mimic several symptoms of AD pathology in a dose- and age-dependent manner. We found in a genetic screen that loss of Polo ameliorated APP-induced AD-like symptoms, presumably by suppressing APP-induced aberrant cell cycle re-entry.

## Results

### Loss of *polo* suppresses APP-induced wing expansion defect

**Figure 1:**
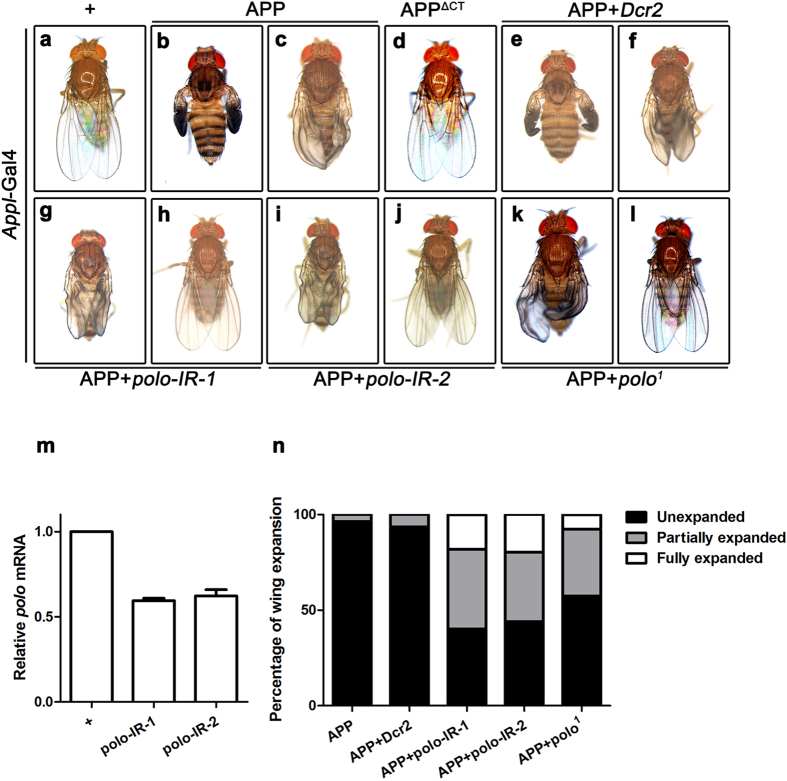
Loss of *polo* suppresses APP-induced wing expansion defect. (**a**–**j**) Light images of adult female flies are shown. Compared with *Appl*-Gal4 controls (**a**), expression of APP (**b** and **c**), but not APP^ΔCT^ (**d**), produced wing expansion defect in adults, almost half of which were suppressed partially or fully by RNAi-mediated down-regulation of *polo* (**g**–**j**) or in heterozygous *polo*^*1*^ mutant (**k**,**l**), but remained unaffected by expressing *UAS*-Dcr2 (**e**,**f**). (**m**) The knocking-down efficiencies of two independent *polo*-RNAi lines are measured by qRT-PCR assay. It was performed on cDNA isolated from brains of 3^rd^ instar larvae for the indicated genotype. (**n**) Statistical analysis of the wing phenotype shown in **b**,**c** and **e**–**l**. The percentage of adult wing with morphological defects is shown. n > 300 for each genotype. The crosses were performed at 25 °C.

To investigate the *in vivo* functions of human APP in animal development, we reasoned the best solution is to express APP in the same pattern as its *Drosophila* homolog APPL, which is expressed primarily in the central nervous system (CNS) in adults and 3^rd^ instar larvae (Figure S1). To this end, we expressed *UAS*-APP under the control of *Appl*-Gal4 (*Appl* > APP), and observed a severe wing expansion defect (96% unexpanded, Fig. 1b; 4% partially expanded, Fig. 1c), as compared with *Appl*-Gal4 controls (Fig. 1a). The wing expansion behavior is regulated by the central nervous system whose dysfunction results in wing expansion deficit^39^. Intriguingly, expression of two truncated APP proteins with deletions in the C-terminal intracellular domain (AICD), APP^ΔCT^ (a truncated form of APP lacking AICD) and APP^ΔNPTY^ (APP with a deletion of the NPTY motif in AICD), failed to recapitulate the wing defect (Fig. 1d and Figure S2b), indicating that AICD (or more specifically, the NPTY motif) is indispensable for APP to impede wing expansion. This result is in agreement with the recent findings that AICD is necessary for APP-induced neuronal cell death in *Drosophila*^40^,^41^.

To identify additional factors that regulate APP functions *in vivo*, we performed a genetic screen in *Drosophila* for *UAS*-RNAi lines that could dominantly suppress APP-induced wing phenotypes. We found that expression of two independent *polo* RNAi lines could partially suppress APP-induced wing defect *(polo-IR*-1, 42% partially expanded and 18% fully expanded; *polo-IR*-2, 37% partially expanded and 20% fully expanded, Fig. 1g–j,n). The knockdown efficiencies of the two *polo*-RNAi lines were verified by quantitative real-time PCR (qRT-PCR, Fig. 1m). To rule out the possibility that the effect of *polo*-RNAi on APP-induced phenotype is a result of Gal4 titration by another UAS sequence, we co-expressed *UAS*-Dicer2 (*UAS*-Dcr2) along with *UAS*-APP, and found that *UAS*-Dcr2 could not alter APP-induced wing defect (94% unexpanded and 6% partially expanded, Fig. 1e,f,n). To further confirm the role of *polo* in APP-induced wing defect, we introduced *polo*^*1*^, a hypomorphic *polo* allele^42^, into *Appl* > APP background. We found that APP-induced wing defect was partially suppressed in heterozygous *polo*^*1*^ mutants (35% partially expanded and 8% fully expanded, Fig. 1k,l,n). Together, these data suggest that Polo is necessary for APP to impede wing expansion.

As Plk1 level is elevated in brain tissues of AD patients^32^,^33^, we wondered whether Polo expression was altered by expressing APP in *Drosophila*. To this end, we first examined *polo* mRNA in 3^rd^ instar larval brain by *in situ* hybridization and qRT-PCR assay. We found *polo* mRNA level was not significantly affected by expressing APP (Figures S3a, S3b and S4). We next checked Polo protein level monitored by a GFP-Polo reporter^43^, and found GFP-Polo fusion protein was not considerably upregulated in the eye disc posterior to the morphogenetic furrow upon APP expression driven by *GMR*-Gal4 (Figures S3e and S3f). On the other hand, both *polo* mRNA expression in the brain and GFP-Polo expression in the eye disc were significantly downregulated by expressing two *polo* RNAi (Figures S3c, S3d, S3g and S3h), which validate the knockdown efficiencies of RNAi lines. Since Polo is a key component of the spindle assembly checkpoint (SAC) pathway^29^,^44^, we also examined whether overexpression of APP could affect the expression of other SAC pathway components. We performed qRT-PCR assay and found that *aurA*, *aurB* and *msp1* expression in the brain were not significantly changed upon APP expression (Figure S4). Together, these results suggest that expression of APP has no effect on the expression of Polo or other components of the SAC pathway.

### Loss of *polo* suppresses APP-induced larval NMJ and locomotion defects

**Figure 2:**
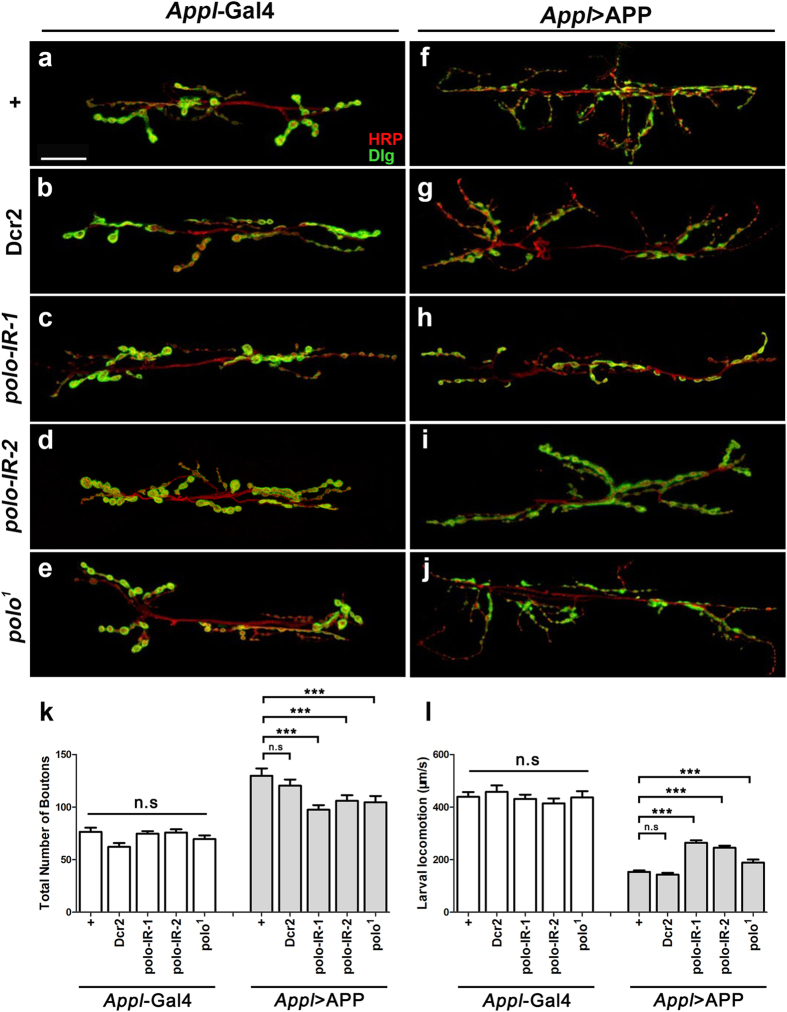
Loss of *polo* impedes APP-induced larval NMJ and locomotion defects. (**a**–**j**) Confocal images of the synapse at segment A3, muscle 6/7 stained with HRP (red) and Dlg (green). Compared with controls (**a**), expression of Dcr2 (**b**) or two independent *polo*-RNAi lines (**c,d**), or heterozygous *polo*^*1*^ mutant (**e**) did not significantly influence the NMJ phenotype. Expression of APP (*Appl* > APP) resulted in an increase of branch and total bouton number (**f**), which was suppressed by RNAi-mediated knocking-down of *polo* (**h**,**i**) or in heterozygous *polo*^*1*^ mutant (**j**), but remained unaffected by expressing Dcr2 (**g**). Scale bar represents 25 μm. (**k**) Statistical analysis of total bouton number per muscle area in **a**–**j** is shown. Error bars mean + S.E.M. ***P < 0.001, n.s, not significant. One-way ANOVA Turkey’s multiple comparison test was used to determine significance between multiple different genotypes. n > 25. (**l**) APP expression induced a crawling deficit in 3^rd^ instar larvae as compared with *Appl*-Gal4 controls. Knocking down *polo* significantly rescued the crawling deficits in APP-expressing larvae, but did not affect the crawling ability in control larvae. One-way ANOVA Turkey’s multiple comparison test was used to determine significance between multiple different genotypes. Error bars mean + S.E.M. ***P < 0.001, n.s, not significant. The crosses were performed at 25 °C.

The *Drosophila* neuromuscular junction (NMJ) is an excellent model for studying synapse formation and function^45^,^46^,^47^. Previous studies reported that expression of APP or APPL in the motor neurons disrupted axonal transport and increased the number of boutons^48^,^49^,^50^,^51^,^52^. We expressed APP specifically under the control of *Appl*-Gal4 and examined the synapse of larval segment A3, muscle 6/7. We observed a significant increase in the total number of boutons and branches at the NMJ in APP-expressing larvae (Fig. 2a,f,k and Figure S5a). To determine whether loss of *polo* could suppress the synaptic defects at the NMJ, we introduced *polo* RNAi or *polo*^*1*^ into *Appl* > APP background. Depletion of *polo*, although exhibited no discernable effect on NMJ synapse morphology (Fig. 2c–e,k and Figure S5a), significantly suppressed APP-induced increased number of boutons and branches at NMJ (Fig. 2h–k and Figure S5a). The type I boutons formed on larval muscles 6/7 could further be subdivided into type I big (1b) and type I small (1s) boutons^53^, which differ not only in their structural properties, but also in their functional properties. We found that the number of type 1b bouton was significantly reduced, whereas that of type 1s increased in *Appl* > APP larvae (Figures S5b and S5c). Both phenotypes were partially suppressed by depletion of *polo*, which by itself had no effect (Figure S5b and S5c).

NMJs are functionally associated with larval locomotion ability. Consistent with the NMJ phenotype, expression of APP driven by *Appl*-Gal4 dramatically reduced the crawling speed of larvae, while loss of *polo* ameliorated this APP-induced larval locomotion defects (Fig. 2l). As a negative control, expression of Dcr2 did not suppress APP-induced larval NMJ and locomotion abnormalities (Fig. 2g,k,l and Figure S5). Together, these results indicate that Polo is required for APP-induced larval NMJ and locomotion defects.

### Loss of *polo* suppresses APP-induced adult locomotion defects

**Figure 3:**
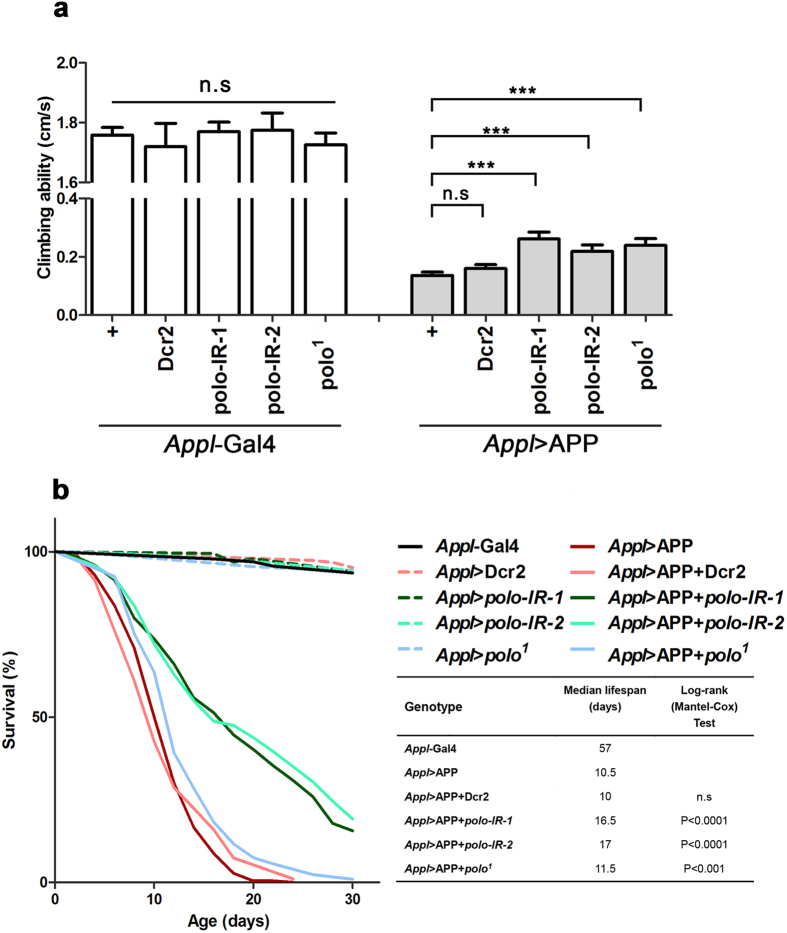
Loss of *polo* ameliorates APP-induced locomotion defect and shortened lifespan in adults. (**a**) Compared with *Appl*-Gal4 controls, *Appl* > APP-induced adult climbing deficit was significantly rescued by loss of *polo* (*Appl* > APP + *polo-IR-1*, *Appl* > APP + *polo-IR-2* and *Appl* > APP + *polo*^*1*^), but not the expression of Dcr2. One-way ANOVA Turkey’s multiple comparison test was used to determine significance between multiple different genotypes. Error bars mean + S.E.M. ***P < 0.001, n.s, not significant. The number of flies tested per genotype was n > 180. The crosses were performed at 25 °C. (**b**) Compared with *Appl*-Gal4 controls, expression of APP resulted in a drastically shortened lifespan, which was suppressed by loss of *polo*. The percentage survivorship was plotted against age. Reported p values comparing median lifespans in the right table are from Mantel-Cox log-rank statistical analysis. The number of flies tested per genotype was n > 210. The crosses were performed at 25 °C.

Negative geotaxis (climbing) assay has been extensively applied in *Drosophila* to assess nervous system dysfunction in studying neurodegenerative diseases^38^. We found that expression of APP driven by *Appl*-Gal4 (*Appl* > APP) resulted in a climbing deficit in adult flies (Fig. 3a). While *Appl*-Gal4 control flies were able to climb at a rate of 1.7 cm/second, *Appl* > APP flies showed a dramatic reduction to less than 0.2 cm/second (Fig. 3a). We found that expression of *polo* RNAi but not Dcr2, and heterozygosity of *polo*^*1*^, could slightly but significantly suppress APP-induced climbing deficit (Fig. 3a). As a control, depletion of *polo* by RNAi or mutation had no apparent effect on flies’ climbing ability (Fig. 3a). Thus, loss of *polo* improves the locomotor dysfunction caused by APP expression.

### Loss of *polo* rescues APP-induced adult lifespan shortening

Lifespan assay provides a statistically robust test of the neurological integrity of a fly. Previous studies reported that expression of human neurodegenerative disease genes would reduce lifespan of *Drosophila*^34^,^36^,^54^. Consistently, expression of APP driven by *Appl*-Gal4 resulted in a drastically shortened lifespan, which was partially rescued by depletion of *polo*, but remained unaffected by expressing Dcr2 (Fig. 3b). Together, these results suggest that loss of *polo* rescues lifespan of APP-expressing flies.

### Loss of *polo* attenuates adult specific APP-induced locomotion and lifespan defects

**Figure 4:**
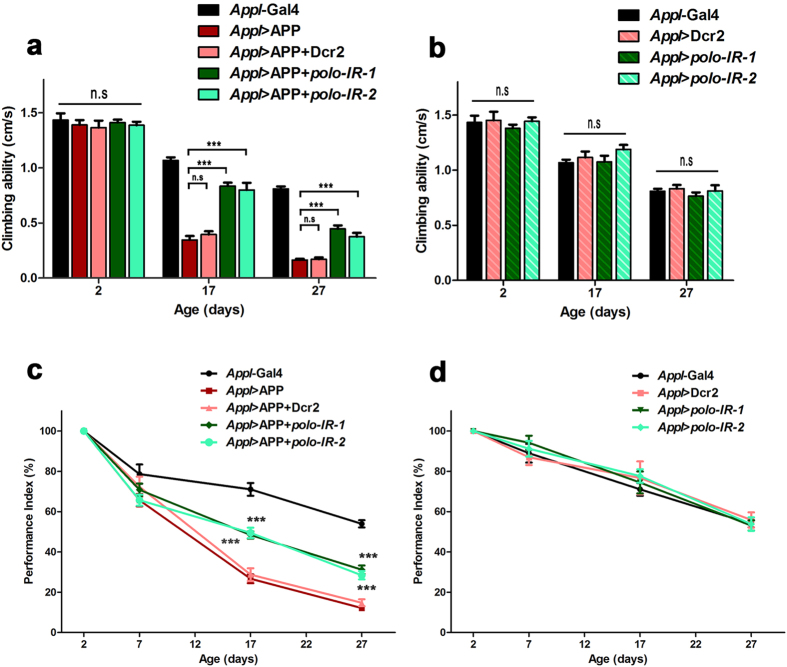
Loss of *polo* attenuates adult-specific APP-induced locomotion decline. (**a**) Adult-specific expression of APP (*Appl* > APP) had no discernable effect on climbing ability in 2-day old flies, but displayed a climbing deficit in 17- and 27-day old flies, as compared with *Appl*-Gal4 controls. Knocking down *polo* significantly rescued APP-induced climbing deficits. (**b**) Knocking down *polo* did not affect the climbing ability in control flies at different time-points. (**c**) Compared with controls (*Appl*-Gal4), *App*l > APP flies displayed a much severer age-dependent decline of climbing ability, which was ameliorated by knocking-down *polo*. (**d**) Knocking down *polo* did not affect the age-dependent decline of climbing ability in control flies. Flies were raised at 17 °C and shifted to 25 °C after eclosion. One-way ANOVA Turkey’s multiple comparison test was used to determine significance between multiple different genotypes. Error bars mean + S.E.M. ***p < 0.001, n.s, not significant. The number of flies tested per genotype was n > 150.

Given that AD is an age-related disease, to better study the pathological functions of APP in adult neurodegeneration, we sought to express APP specifically in the adult stage, thus avoiding its earlier developmental effects. Since the Gal4/UAS system is sensitive to temperature, to inhibit APP expression in earlier development, we raised the embryos at a lower temperature (17 °C) throughout the larva and pupa stages, and shifted the freshly eclosed flies to 25 °C to allow the expression of APP in adulthood. Although *Appl* > APP flies reared at 25 °C displayed abnormal wing phenotype (Fig. 1b,c), those raised at 17 °C did not produce any obvious morphological defects (Figures S6a and S6b). In addition, no apparent climbing defect was observed in 2-day old *Appl* > APP flies, as compared with the controls (Fig. 4a). These data suggest that the developmental effects of APP were effectively avoided.

Previous studies suggested that flies’ locomotion capability diminishes with aging^37^,^54^,^55^,^56^. Consistently, we observed a decreased climbing ability in control flies at 17 days and 27 days, respectively (Fig. 4a). Interestingly, the climbing ability of APP-expressing flies declined more dramatically with aging (Fig. 4a), indicative of an age-related neurodegeneration resulted from adult-specific expression of APP. Expressing *polo* RNAi, but not Dcr2, considerably rescued the locomotion defect in APP-expressing flies at the corresponding age points (Fig. 4a), though loss of *polo* by itself did not affect flies’ climbing ability as compared with age-matched controls (Fig. 4b).

To accurately quantify the development of age-dependent locomotion disability, we defined a performance index (PI) - the relative decline of the climbing ability between aged and young (2-day old) flies in a climbing assay: PI = velocity_aged_/velocity_young_. With aging, the *Appl*-Gal4 control group displayed a gentle decline in the climbing ability (Fig. 4c), whereas APP-expressing flies began to display a miniscule but statistically significant locomotor dysfunction at 7-day old, which became more obvious and drastic at 17- and 27-day old (Fig. 4c). Knocking-down *polo* significantly ameliorated the decline of locomotor function in APP-expressing flies (Fig. 4c), but did not affect the climbing ability in control flies (Fig. 4d).

We also checked the effect of adult-specific expression of APP on lifespan at 29 °C. Consistent with the locomotion disability, APP expression resulted in a significantly shortened lifespan, which was partially rescued by knocking-down *polo*, but remained unchanged by expressing Dcr2 (Figure S7). These results indicate that loss of *polo* could ameliorate adult-specific APP-induced locomotor dysfunction and lifespan shortening.

### Loss of *polo* ameliorates APP-induced male courtship choice defect

**Figure 5:**
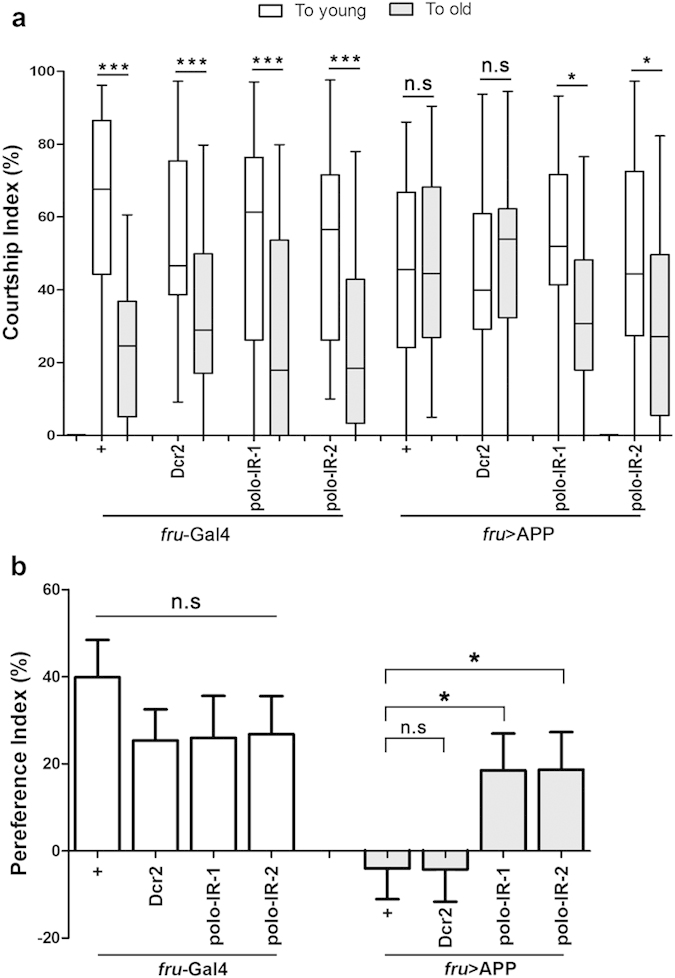
Loss of *polo* restores APP-induced male courtship choice disorder. (**a**) Courtship index of 3-day-old naive males in choice assays toward younger virgin females (white) and older ones (grey) are shown. The courtship index of control males (*fru*-Gal4) to younger virgin females is much higher than their courtship index to older ones. Expression of APP in male courtship circuit (*fru* > APP) eliminates male’s courtship preference to younger mates, while knocking down *polo* restores the choice preference. (**b**) Preference index of 3-day-old naive males in courtship choice assays. Mean ± standard error of the mean (SEM), n > 28 for each genotype; Kruskal-Wallis test, Dunn's post-hoc was used to determine significance for each comparison. ***p < 0.001, *p < 0.05, n.s, not significant. The crosses were performed at 25 °C. Box-and-whisker plots for CIs show 1–99 percentiles and means (+), n > 28 for each genotype; Wilcoxon matched pairs test was used to determine significance between intragroup courtships toward younger females and older ones.

Cognitive decline is another critical hallmark of AD^1^. Male courtship preference behavior, a paradigm for decision making in animals, to a certain degree, reflects the discriminative and cognitive ability. Previous study reported that *Drosophila* males, when provided with both younger and older virgin females, tended to be fastidious and preferred to court younger ones^57^. Interestingly, this courtship preference was eliminated in aged males or males expressing APP in the courtship specific neurons driven by *fru*-Gal4^57^, suggesting that expression of APP in *Drosophila* could mimic aging-induced choice disorder and cognitive impairment. Consistent with the previous report, we found 3-day old *fru*-Gal4 control males preferred younger virgin females to older ones, while *fru* > APP males were unable to distinguish younger females from older ones (Fig. 5a). Knocking-down *polo* did not influence males’ courtship preference behavior, but ameliorated APP-induced male choice dysfunction and partially restored their courtship preference to younger mates (Fig. 5a).

To accurately quantify the extent of a male’s preference for younger or older females, we also checked the preference index (PI) that indicate the relative difference between male’s courtship percentage toward younger females and that toward older ones in a choice assay: PI = (CIy-CIo)/(CIy + CIo)^57^. Consistently, the PI of APP-expressing flies drastically dropped compared with that of controls, and was significantly suppressed by knocking down *polo* (Fig. 5b). Thus, these results indicate that Polo is also required for APP-induced cognitive disability.

### Loss of *polo* impedes APP-induced retinal degeneration and cell cycle re-entry

**Figure 6:**
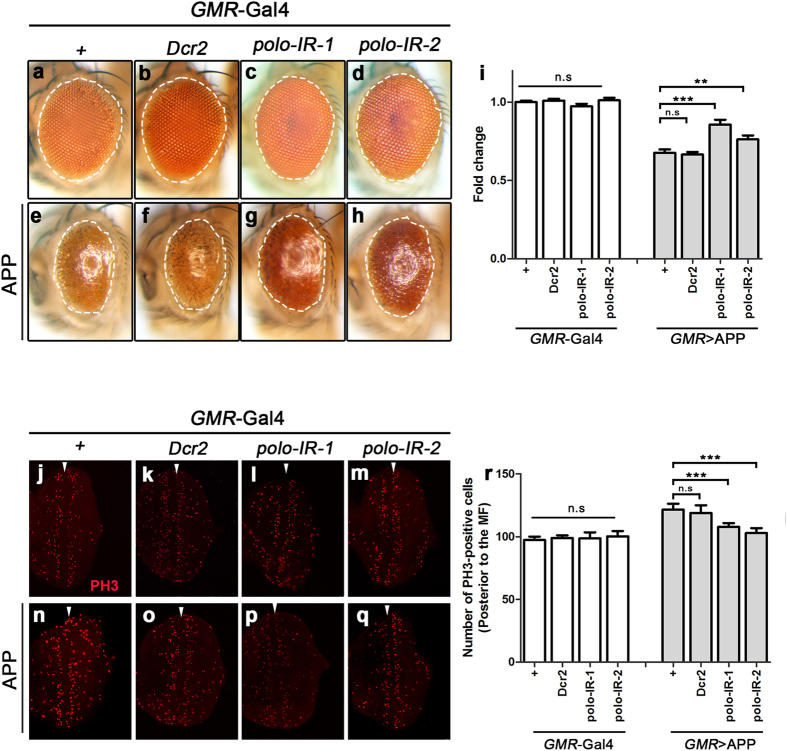
Loss of *polo* suppresses APP-induced retina degeneration and cell cycle re-entry. (**a**–**h**) Light images of *Drosophila* adult eyes are shown. The white dashed lines outline the eye contour. Compared with the control (**a**,**b**), expression of APP (*GMR* > APP) induced small and rough eyes with reduced pigmentation (**e**). Loss of *polo* did not affect eye development (**c**,**d**), but suppressed APP-induced retina degeneration (**g,h**). (**i**) Statistical analysis of eye size shown in (**a**–**j**). The fold change of eye size compared with control is shown. The size of adult eye from control group (*GMR*-Gal4) was normalized to a value of 1. "Fold change" represents the size of adult eye from indicated genotype normalized with the control. One-way ANOVA Turkey’s multiple comparison test was used to determine significance between multiple different genotypes. Error bars mean + S.E.M. ***P < 0.001, n.s, not significant, n > 15 for each genotype. The crosses were performed at 25 °C. (**j**–**q**) Fluorescent images of 3^rd^ instar larval eye discs stained with phospho-histone 3 (PH3) showing the number of mitotic cells in M phase. White arrowheads indicate the morphogenetic furrow (MF). Compared with the control (**j**,**k**), reduction of *polo* did not affect the PH3 level (**l**,**m**). Expression of APP significantly upregulated the level of PH3 posterior to MF (**n**), which was suppressed by loss of *polo* (**p**,**q**). (**r**) Statistical analysis of mitotic cell number posterior to the MF in (**j**–**q**). The number of PH3-positive cells posterior to the MF is shown. One-way ANOVA Turkey’s multiple comparison test was used to determine significance between multiple different genotypes. Error bars mean + S.E.M. ***P < 0.001, n.s, not significant, n > 15 eye discs for each genotype. The crosses were performed at 25 °C.

Previous work suggested that expression of human neurodegenerative disease genes induced neurotoxicity in fly eye^58^,^59^,^60^. To investigate the mechanism by which loss of *polo* ameliorates APP-induced neurotoxicity, we drove APP expression in developing eyes by the *GMR*-Gal4 driver^61^. We found that pan-retinal expression of APP resulted in eye deterioration characterized by small and rough eyes with reduced pigmentation (Fig. 6a,e,i). Although knocking-down *polo* alone had no distinguishable effect on eye development (Fig. 6c,d,i), it partially suppressed APP-induced retina degeneration (Fig. 6g–i), suggesting Polo is indispensable for APP-induced retina toxicity in adult eyes.

Given that Polo is a key regulator of cell cycle, and aberrant cell cycle-triggered cell death has been associated with neuronal degeneration in AD brain^18^, we wondered whether abnormal cell cycle re-entry is involved in *GMR* > APP-induced eye degeneration. To address this issue, we examined cell cycle state of third instar larval eye discs. We incubated eye imaginal disc with Bromo-2′-deoxyuridine (BrdU) to ascertain whether APP could induce cells re-entering S phase or re-synthesizing DNA. Compared with control eye discs, expression of APP by *GMR*-Gal4 significantly increased the number of cells incorporated BrdU (Figure S8). Since previous study also reported that the level of phospho-histone H3 (PH3), a marker of M phase, increased in hippocampal neurons in AD patients^62^, we wondered whether expression of APP could further promote cells to go through the G2/M transition into M phase. In control eye discs, only a few scattered cells were labeled with PH3 posterior to the second mitotic wave (SMW), suggesting that most cells posterior to SMW had exited the cell cycle (Fig. 6j)^63^. Expression of APP posterior to the morphogenetic furrow (MF) driven by *GMR*-Gal4 increased the number of PH3 + cell in the posterior part of eye discs (Fig. 6n,r), suggesting that expression of APP promoted cells re-entering cell cycle to M phase. Knocking-down *polo* significantly suppressed *GMR* > APP-induced increase of PH3 + cells (Fig. 6p–r), suggesting that loss of *polo* is able to abrogate APP-induced neurotoxicity by preventing aberrant cell cycle re-entry. These results likely suggest that knocking-down *polo* partially suppresses APP-induced retinal degeneration by preventing APP-induced cell cycle re-entry.

## Discussion

*Drosophila* has emerged as an excellent animal model to study human neurodegenerative diseases^36^,^41^,^64^,^65^. Ectopic expression of AD-related proteins (various isoforms of Tau proteins, Aβ peptides and APP) in flies produced a number of phenotypes, which greatly contributed to the understanding of their *in vivo* functions and the pathogenesis of AD^18^,^34^,^38^,^41^,^50^,^54^. APP belongs to an evolutionary conserved protein family that also includes the mammalian APLP1 and APLP2, the *Drosophila* APPL, and the *C. elegans* APL-1^66^. APPL was reported to be exclusively expressed in the nervous system in embryonic stages^67^, yet its expression in later stage remains elusive. Here, using *UAS*-GFP to track the expression pattern of *Appl*-Gal4, we found that *Appl* is also predominantly expressed in the larval and adult CNS (Figure S1). Thus, compared with previous studies expressing APP pan-neuronally by *elav*-Gal4, here we generated a fly AD model by expressing APP in the same pattern as *Appl*. Based on this model, we performed a genetic loss-of-function screen to search dominant modifiers of ectopic APP-induced phenotypes. Genes identified from the screen presumably function in *Appl*-expressing neurons, and are more likely to interact with *Appl* in development.

Interestingly, we found that expression of APP driven by *Appl*-Gal4 caused wing expansion defect. As an insect that would undergo complete metamorphosis, in *Drosophila*, the hardening of the expanded wings marks the end of morphological development^68^,^69^,^70^. Previous studies reported that the wing-expansion program is governed by neuroendocrine networks^39^,^70^,^71^, for instance, the neurohormone bursicon functions within the *Drosophila* CNS to modulate wing expansion behavior^39^. In addition, the network underlying wing expansion has been shown to include a dozen of neurons implicated in both larval and pupal ecdysis in *Drosophila*^72^,^39^,^73^,^74^,^75^. For example, suppression of excitability of bursicon-expressing neurons or ablation of CCAP-expressing neurons by cell death gene during development would lead to a severe wing expansion deficit in adult flies^39^,^73^,^76^. Accordingly, expression of APP by *Appl*-Gal4 likely caused malfunction or cell death in the CNS, resulting in the wing expansion deficits. Interestingly, AICD is necessary for APP to generate this phenotype (Fig. 1d). This result is in agreement with recent findings that AICD is required for APP-induced cell death in *Drosophila*^40^,^41^.

Polo kinase is one of the best characterized Ser/Thr protein kinase. As a key mitotic regulator, Polo kinase governs multiple aspects of mitosis including mitotic entry, centrosome maturation, spindle formation, chromosome segregation, and cytokinesis^23^,^24^,^25^,^26^,^27^,^28^,^29^,^30^,^31^. Recent studies reported that Polo was involved in activation of the anaphase-promoting complex/cyclosome (APC/C) and allowed mitotic exit^29^,^44^. Genetic and biochemical studies have shown that Polo plays crucial role in controlling Spindle Assembly Checkpoint (SAC) pathway to keep the maintenance of genomic stability during mitosis, and is required for correct localization of AurB and promotes recruitment of Mps1^29^,^44^.

Intriguingly, Plk1, the homolog of Polo, is highly present in susceptible hippocampal and cortical neurons of AD patients compared with age-matched controls^32^. However, the exact role of Plk1 in the pathology of AD has remained unclear. In this study, we examined both mRNA and protein expression levels of *polo*, and found that overexpression of APP did not significantly affect the expression of *polo* (Figures S3 and S4). In addition, we did not observe any obvious alteration on the expression of *aurB* and *msp1* (Figures S4), which encode important components of SAC pathway. Thus, up-regulation of Plk1 in neurons of AD patients may not be a direct outcome of elevated APP activities.

In the present study, we expressed human APP, an AD causative gene, in the same temporal-spatial pattern as its *Drosophila* homolog Appl. By taking advantage of the temperature-dependent activity of the Gal4/UAS system, we raised flies at different temperatures to investigate the pathological outcomes of APP expression in development or adult-onset. We demonstrated that expression of APP resulted in morphological defect in adult wings, synaptic abnormalities in larval NMJ, locomotion decline, male choice dysfunction and shortened lifespan. Some of the phenotypes resemble AD-like symptoms. Genetic depletion of *polo* ameliorated APP-induced morphological and behavioral defects. Finally, loss of *polo* suppressed APP-induced eye degeneration and cell cycle re-entry. These findings not only help us gain further understandings of the physiological and pathological functions of APP, but also shed light on discovering a potential cure or prevention of AD.

## Materials and Methods

### Fly Strains

Unless otherwise indicated, flies were kept on a cornmeal and agar medium at 25 °C according to standard protocols, and the data are depicted from female flies.

*Drosophila* strains used include: *UAS*-APP, *UAS*-APP695^ΔCT^ and *Appl*-Gal4^40^, *GMR*-Gal4^61^, *Frutless*-Gal4^57^ and GFP-Polo^43^ were previously described. *UAS*-APP^ΔNPTY^ (29869), *UAS-polo-IR-1* (33042), *UAS-polo-IR-2* (35146), *UAS*-Dcr2 (24651), *UAS*-GFP (32198) and *polo*^*1*^ (546) were obtained from the Bloomington *Drosophila* Stock Center.

### Behavioral testing

For behavioral experiments, the *Appl*-GAL4 line was outcrossed with *w*^*1118*^ line for eight generations.

For larvae crawling assay, the procedures were performed as described^40^. 3^rd^ instar larvae were collected and rinsed in 1% PBS, and subsequently transferred onto 3% agarose plate. Larvae were allowed to rest for 3 min before videotaping. The locomotion behavior was analyzed by the Nikon software (NIS-Elements D, Nikon). Each genotype was tested with five larvae and each larva was tested for five times. For the track of the larvae, each genotype was recorded for 10 min and repeated for five times.

For Rapid Iterative Negative Geotaxis (climbing) assay, a modified version of Nichols was used^77^. Briefly, Flies were collected within 24 hours after eclosion. 25 ~ 30 flies were placed in a vertical vial (20 cm height, 2.5 cm diameter) and tapped to the bottom of the vial. After 5 seconds, a picture was taken and the average height climbed was then recorded. Velocity defined as (height/5s) was calculated. Each analysis was repeated 4 times with 60 seconds resting interval. The number of flies tested per genotype was n > 100. For Fig. 3, the analysis was performed 2 days post-eclosion (d.p.e.). For Fig. 4, the flies were raised at 17 °C and shifted to 25 °C after eclosion. The analysis was tested serially for 2 ~ 27 d.p.e. The performance index (PI) was defined as Velocity_n d.p.e._/Velocity_2d.p.e_.

For lifespan analysis, flies were collected within 24 hours after eclosion and placed in a food vial at a density of 20 ~ 25 flies per vial, transferred to fresh vials every 2 to 3 days, and dead flies were counted at that time. The number of flies tested per genotype was n > 200. Data are presented as survival curves and analysis was performed using log-rank tests to compare between groups.

For male courtship choice assay, the procedures were performed as described^57^. Courtship behavior assays were performed at approximately the same time each day (within 1 hour at the beginning of the 12-hour illumination half of the cycle) in round observation chambers (1.5 cm in diameter and 0.3 cm deep) by observing courtship behavior of males (3-day old). Choice-assays were performed by pairing a naive male with 2 younger (3 ~ 5-day old) wild type virgin females and 2 older (30 ~ 35-day old) ones. All tests were recorded for 10 minutes with an HDR-CX270 digital video camera (Sony) and analyzed with Noldus EthoVision XT software (Noldus Information Technology). The courtship index (CI) was calculated as the percentage of time that a male courted the females during a 10-minute period. The preference index (PI) defined as (CIy-CIo)/(CIy + CIo) was calculated. The number of male flies tested per genotype was n > 28.

### Immunohistochemistry and BrdU labelling

Eye discs were dissected from 3^rd^ instar larvae in cold PBS and fixed in 4% paraformaldehyde. After proper washes, the discs were blocked in 10% horse serum and stained with primary antibody (rabbit anti-PH3, Sigma). Subsequently, fluorescence conjugated secondary antibodies were used for signal detection.

For BrdU labelling^78^, eye discs were dissected from 3rd instar larvae in PBS, incubated with 200 μg/ml BrdU (Sigma) in Schneider’s media for 40 min then fixed in 5% formaldehyde, then washed in PBST and hydrolyzed in 2 N HCl. After proper washes, the discs were blocked in 10% horse serum and stained with primary antibody (mouse anti-BrdU, Becton Dickinson); subsequently, fluorescence conjugated secondary antibodies were used for signal detection.

For NMJ, 3^rd^ instar larvae were dissected in PBS and fixed in 4% paraformaldehyde for 20 minutes and washed with PBS containing 0.3% Triton X-100 (PBT). Larval body walls were stained overnight with primary antibody (anti-Dlg and HRP-TRITC) in 1% normal donkey serum (NDS) in PBT at 4 °C, followed by 3 washes in PBT, 2 hours incubation with secondary antibody and Phalloidin-TRITC diluted in 1% NDS in PBT at room temperature, 4 washes with PBT. Larval body walls were mounted in Vectashield (Vector Labs, H-1000). Confocal images were collected from Leica confocal microscope SP5 equipped with an oil immersion objective (X63 HCX PL APO 1.25). Leica Application Suite Advanced Fluorescence software was used to capture, process and analyze images.

### *In Situ* Hybridization

*In situ* hybridization was performed on larvae brains as previously described^79^. Digoxigenin (DIG)-labeled (Roche) *polo* RNA probes were generated by transcribing plasmid pGEM-T Easy, with SP6 polymerase (antisense probe) or with T7 polymerase (sense probe), respectively.

### Quantitative Real Time RT-PCR

Total RNA was isolated from the brains of wandering third instar larvae from different genotypes using TRIzol reagent (Invitrogen). n > 60 brains of indicated larvae were collected. RT-PCR was performed as previously described^80^. It was performed in triplicate and the fold change was calculated by using the ΔΔCT method using RP49 as control. The results from 4 independent tests. Primers for *polo* are kindly provided by Dr. Lipsick at Stanford University^81^. Primers for *Rp49* are kindly provided by Dr. Ketu Mishra at Yale University. The primers for the target genes analyzed are as follows: for aurB, forward primer 5′TGATGTTCAAAGAGGAGCTGC 3′ and reverse primer 5′-CTCGTCGTGGAACCAAGTGAG-3′; msp1, forward primer 5′ -AGGACGACTTTAACACGCCAT-3′ and reverse primer 5′ -CCCTCGTTTGGACTTGGAAAGA-3′; aurA, forward primer 5′ -AGCCCAACAGCGAGAATATGG-3′ and reverse primer 5′ -GGAAGCTATGGAATTGGAGCCT-3′

## Additional Information

**How to cite this article**: Peng, F. *et al.* Loss of Polo ameliorates APP-induced Alzheimer's disease-like symptoms in *Drosophila*. *Sci. Rep.* **5**, 16816; doi: 10.1038/srep16816 (2015).

## Supplementary Material

Supplementary Information
