# Supplementary material for: Loss of Polo ameliorates APP-induced Alzheimer’s disease-like symptoms in Drosophila
Source: Sci Rep. 2015 Nov 24;5:16816. doi: 10.1038/srep16816 (PMC4657023; doi:10.1038/srep16816)

Loss of Polo ameliorates APP-induced Alzheimer's disease-like symptoms in *Drosophila*

Fei Peng[1], Yu Zhao[1], Xirui Huang[1], Changyan Chen[1], Lili Sun[2], Luming Zhuang[1] and Lei Xue[*1]

Supplementary Information

Figure S1. Expression pattern of *Appl*-Gal4 *in vivo*

Appl-Gal4 drives *UAS*-GFP expression in the brain of adults (**a**) or 3rd instar larvae (**b**), but not in the eye (**c**) or wing (**d**) imaginal disc of 3rd instar larvae. The crosses were performed at 25°C

Figure S2. The NPTY motif is required for APP to induce wing expansion defect

Light images of adult females are shown. Compared with *Appl*-Gal4 controls (**a**), expression of APP$^{\Delta NPTY}$ did not produce any evident wing phenotype (**b**).

Figure S3. Polo expression is not affected by ectopic APP

(**a-d**) *polo* mRNA expression in 3rd instar larval brain were visualized by *in situ* hybridization using a *polo* antisense RNA. Compared with *Appl*-Gal4 controls (**a**), the mRNA level of *polo* in larval brain was not affected by expressing APP (**b**) , but was significantly reduced by expressing two *polo* RNAi (**c** and **d**). The crosses were performed at 25°C

(**e-h**) *Polo* expression in eye imaginal discs of 3rd instar larvae that were monitored by GFP-Polo. Compared with *GMR*-Gal4 controls (**e**), GFP-Polo expression posterior to the morphogenetic furrow was not altered by expressing APP (**f**) , but was reduced by expressing two *polo* RNAi (**g** and **h**). The crosses were performed at 29°C

Figure S4. Expression of SAC pathway components were not affected by expressing APP

Histogram showing the levels of mRNAs of SAC-related genes (*polo, aurA, aurB* and *mps1*) measured by quantitative RT-PCR. Total RNA was isolated from the brain of wandering 3rd instar larvae. Error bars represent standard deviation from four independent experiments. The crosses were performed at 25°C

Figure S5. Loss of Polo suppresses APP-induced larval NMJ defects

(**a-c**) Statistical analysis of branch and bouton number per muscle area on muscle 6/7 of A3. (**a**) Total number of branches is shown. (**b**) Total number of type I b boutons is shown. (**c**) Total number of type I s boutons is shown. Type I b and I s boutons were determined based on bouton size (the diameter of I b bouton is equal or greater than 3 μm, the diameter of I s bouton is less than 3 μm). Error bars means $\pm$ S.E.M. *** P< 0.001, ** P< 0.01, n.s, not significant. One-way ANOVA Turkey's multiple comparison test was used to determine significance between multiple different genotypes. n>25 for each genotype. All crosses were performed at 25°C

Figure S6. *Appl*>APP flies raised at low temperature display normal wings

Light images of adult flies are shown. When raised at 17°C, *Appl*>APP flies did not display wing defects (**b**), as compared with *Appl*-Gal4 controls (**a**). All crosses were performed at 17°C and the flies were shifted into 25°C after eclosion.

Figure S7. Loss of *polo* ameliorates APP-triggered lifespan shortening

Compared with *Appl*-Gal4 control flies, expression of APP (*Appl*>APP) resulted in a shorter lifespan, which was partially rescued by knocking-down *polo* (*Appl*>APP + *polo-IR-1* and *Appl*>APP + *polo-IR-2*). The percentage survivorship was plotted against age. Reported p values comparing median lifespans in the table are from Mantel-Cox log-rank statistical analysis. Flies were raised at 17°C and shifted to 29°C after eclosion

Figure S8. Expression of APP promotes BrdU staining

Fluorescent images of 3rd instar larval eye discs stained with BrdU showing the number of mitotic cells in S phase. Compared with the control (**a**), expression of APP significantly upregulated the level of BrdU staining posterior to MF (**b**).

Detailed Genotypes

Figure 1

(a) *Appl*-Gal4/+

(b and c) *Appl*-Gal4/+; *UAS*-APP/+

(d) *Appl*-Gal4/+; *UAS*-APP$^{\Delta CT}$/+

(e and f) *Appl*-Gal4/+; *UAS*-APP/+; *UAS*-Dcr2/+

(g and h) *Appl*-Gal4/+; *UAS*-APP/+; *UAS-polo-IR-1*/+

(i and j) *Appl*-Gal4/+; *UAS*-APP/+; *UAS-polo-IR-2*/+

(k and l) *Appl*-Gal4/+; *UAS*-APP/+; *polo*^{1}/+

(m) *Appl*-Gal4/+

Appl-Gal4/+; *UAS-polo-IR-1*/+

Appl-Gal4/+; *UAS-polo-IR-2*/+

Figure 2

(a) *Appl*-Gal4/+

(b) *Appl*-Gal4/+; *Dcr2*/+

(c) *Appl*-Gal4/+; *polo-IR-1*/+

(d) *Appl*-Gal4/+; *polo-IR-2*/+

(e) *Appl*-Gal4/+; *polo*^{1}/+

(f) *Appl*-Gal4/+; *UAS*-APP/+

(g) *Appl*-Gal4/+; *UAS*-APP/+; *UAS-Dcr2*/+

(h) *Appl*-Gal4/+; *UAS*-APP/+; *UAS-polo-IR-1*/+

(i) *Appl*-Gal4/+; *UAS*-APP/+; *UAS-polo-IR-2*/+

(j) *Appl*-Gal4/+; *UAS*-APP/+; *polo*^{1}/+

Figures 3 and 4

(*Appl*-Gal4) *Appl*-Gal4/+

(*Appl*>Dcr2) *Appl*-Gal4/+; *UAS*-Dcr2/+

(*Appl*>*polo-IR-1*) *Appl*-Gal4/+; *UAS-polo-IR-1*/+

(*Appl*>*polo-IR-2*) *Appl*-Gal4/+; *UAS-polo-IR-2*/+

(*Appl*>*polo*^{1}) *Appl*-Gal4/+; *polo*^{1}/+

(*Appl*>APP) *Appl*-Gal4/+; *UAS*-APP/+

(*Appl*>APP+ Dcr2) *Appl*-Gal4/+; *UAS*-APP/+; *UAS*- Dcr2/+

(*Appl*>APP+*polo-IR-1*) *Appl*-Gal4/+; *UAS*-APP/+; *UAS-polo-IR-1*/+

(*Appl*>APP+*polo-IR-2*) *Appl*-Gal4/+; *UAS*-APP/+; *UAS-polo-IR-2*/+

(*Appl*>APP+*polo*^{1}) *Appl*-Gal4/+; *UAS*-APP/+; *polo*^{1}/+

Figure 5

fru-Gal4/+

fru-Gal4/ *UAS*-Dcr2

fru-Gal4/*UAS-polo-IR-1*

fru-Gal4/*UAS-polo-IR-2*

UAS-APP/+; *fru*-Gal4/+

UAS-APP/+; *fru*-Gal4/ *UAS*-Dcr2

UAS-APP/+; *fru*-Gal4/ *UAS-polo-IR-1*

UAS-APP/+; *fru*-Gal4/ *UAS-polo-IR-2*

Figure 6

(a and j) *GMR*-Gal4/+

(b and k) *GMR*-Gal4/+; *UAS*-Dcr2/+

(c and l) *GMR*-Gal4/+; *UAS-polo-IR-1*/+

(d and m) *GMR*-Gal4/+; *UAS-polo-IR-2*/+

(e and n) *GMR*>APP/*UAS*-APP

(f and o) *GMR*>APP/*UAS*-APP; *UAS*-Dcr2/+

(g and p) *GMR*>APP/*UAS*-APP; *UAS-polo-IR-1*/+

(h and q) *GMR*>APP/*UAS*-APP; *UAS-polo-IR-2*/+

Figure S1

Appl-Gal4/+; *UAS*-GFP/+

Figure S2

(A) *Appl*-Gal4/+

(B) *Appl*-Gal4/+; *UAS*-APP$^{\Delta NPTY}$/+

Figure S3

(a) *Appl*-Gal4/+

(b) *Appl*-Gal4/+; *UAS*-APP/+

(c) *Appl*-Gal4/+; *UAS*-APP/+; *polo-IR-1*/+

(d) *Appl*-Gal4/+; *UAS*-APP/+; *polo-IR-2*/+

(e) *GMR*-Gal4/GFP-Polo

(f) *GMR*>APP/ GFP-Polo

(g) *GMR*>APP/ GFP-Polo; *UAS-polo-IR-1*/+

(h) *GMR*>APP/ GFP-Polo; *UAS-polo-IR-2*/+

Figure S4

(a) *Appl*-Gal4/+

(b) *Appl*-Gal4/+; *UAS*-APP/+

Figure S5

Appl-Gal4/+

Appl-Gal4/+; *UAS*-Dcr2/+

Appl-Gal4/+; *UAS-polo-IR-1*/+

Appl-Gal4/+; *UAS-polo-IR-2*/+

Appl-Gal4/+; $polo^{1}$/+

Appl-Gal4/+; *UAS*-APP/+

Appl-Gal4/+; *UAS*-APP/+; *UAS*-Dcr2/+

Appl-Gal4/+; *UAS*-APP/+; *UAS-polo-IR-1*/+

Appl-Gal4/+; *UAS*-APP/+; *UAS-polo-IR-2*/+

Appl-Gal4/+; *UAS*-APP/+; *polo*^{1}/+

Figure S6

(a) *Appl*-Gal4/+

(b) *Appl*-Gal4/; *UAS*-APP/+

Figure S7

(*Appl*-Gal4) *Appl*-Gal4/+

(*Appl*>APP) *Appl*-Gal4/+; *UAS*-APP/+

(*Appl*>APP+Dcr2) *Appl*-Gal4/+; *UAS*-APP/+; *UAS*-Dcr2/+

(*Appl*>APP+*polo-IR-1*) *Appl*-Gal4/+; *UAS*-APP/+; *UAS-polo-IR-1*/+

(*Appl*>APP+*polo-IR-2*) *Appl*-Gal4/+; *UAS*-APP/+; *UAS-polo-IR-2*/+

Figure S8

(a) *GMR*-Gal4/+

(b) *GMR*>APP/*UAS*-APP

Peng et al., Fig. S1

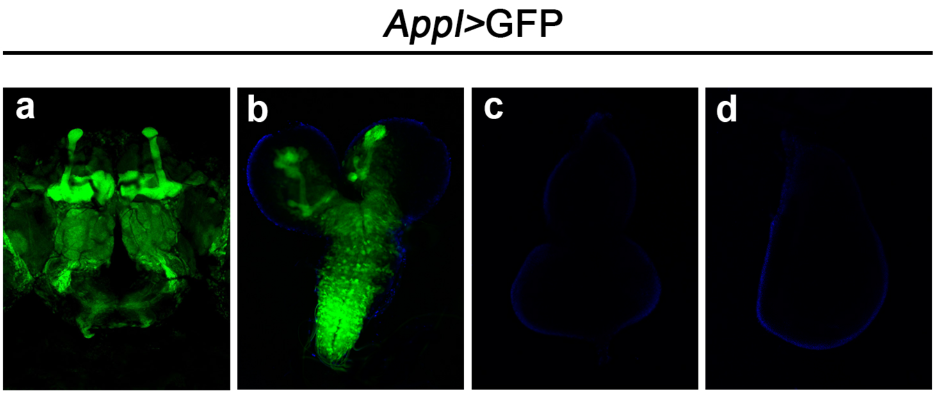

Peng et al., Fig. S2

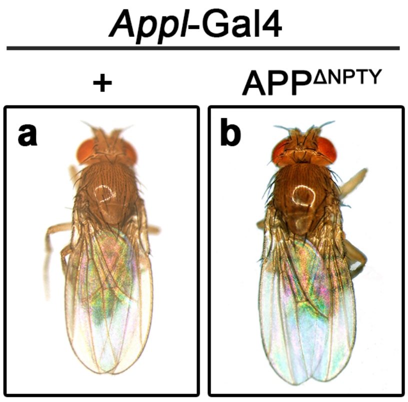

Peng et al., Fig. S3

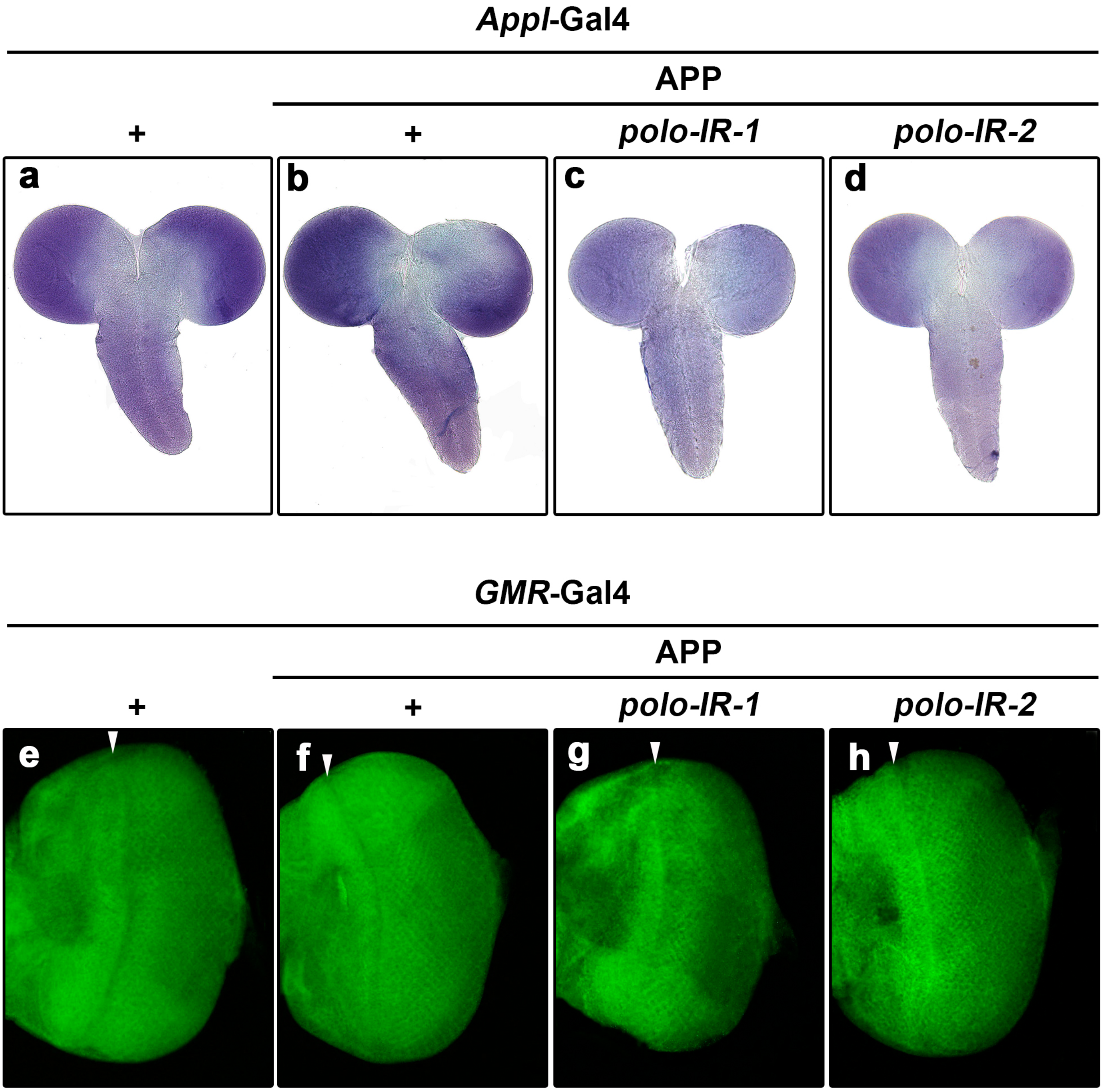

Peng et al., Fig. S4

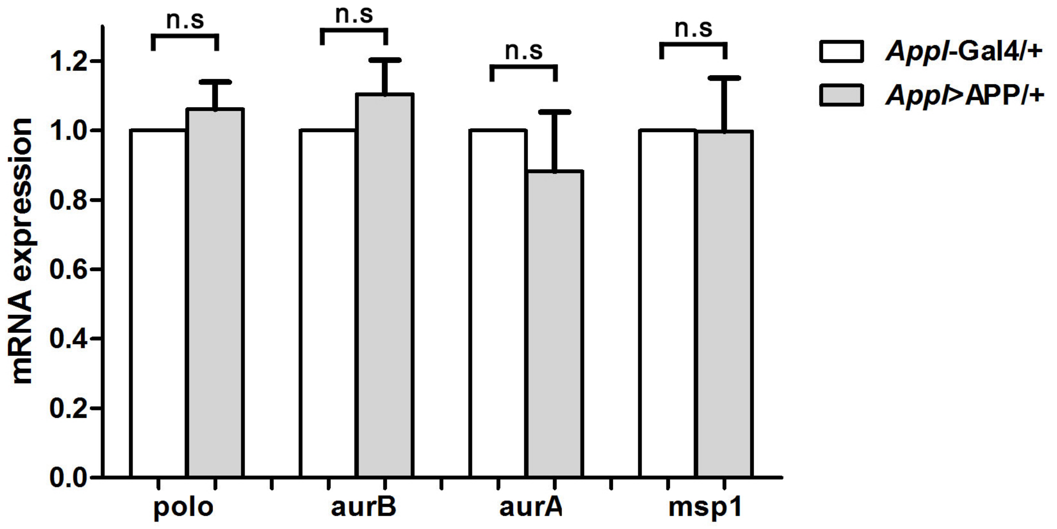

Peng et al., Fig. S5

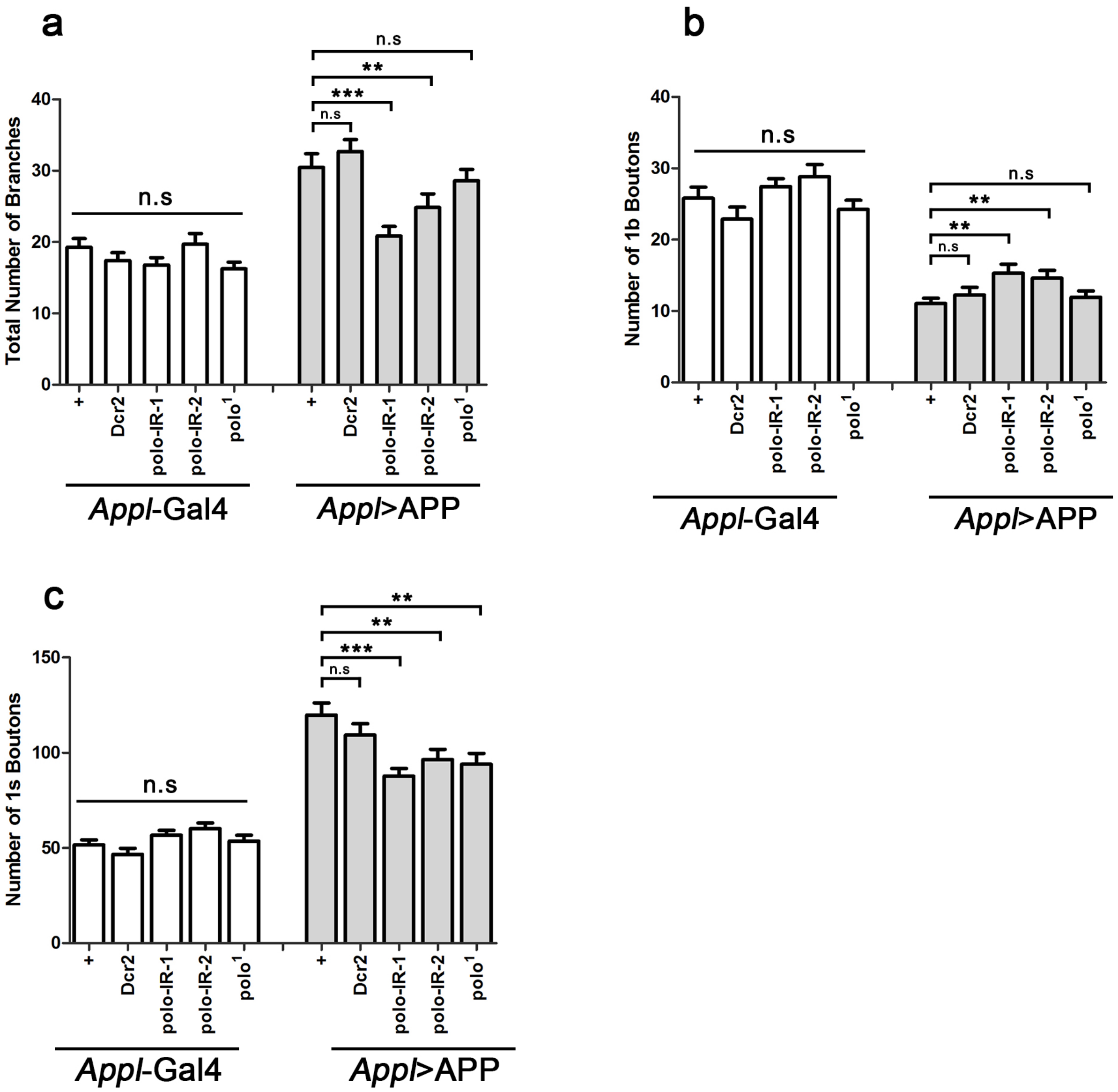

Peng et al., Fig. S6

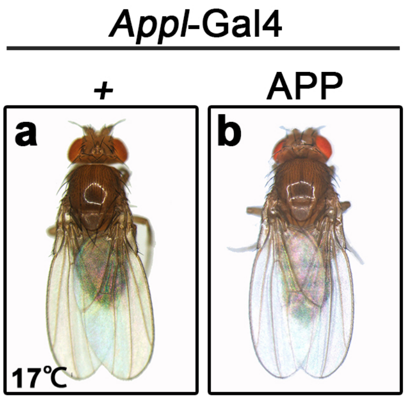

Peng et al., Fig. S7

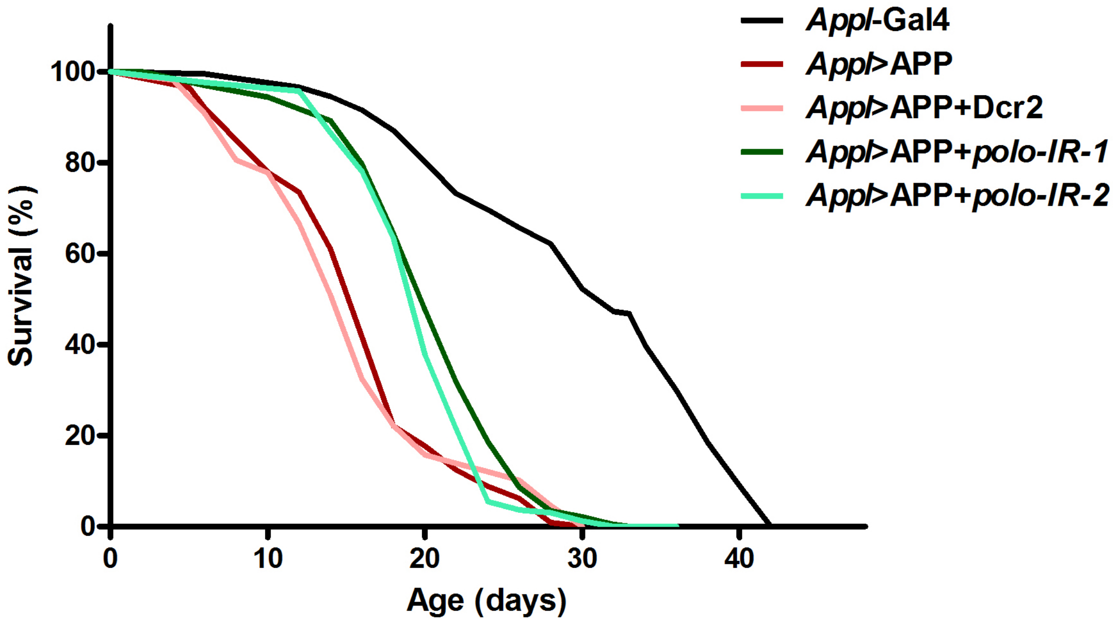

Genotype	Median lifespan (days)	Log-rank (Mantel-Cox) Test
Appl-Gal4	32	
Appl>APP	16	
Appl>APP+Dcr2	15.5	n.s
Appl>APP+*polo-IR-1*	20	P<0.0001
Appl>APP+*polo-IR-2*	20	P<0.0001

Peng et al., Fig. S8

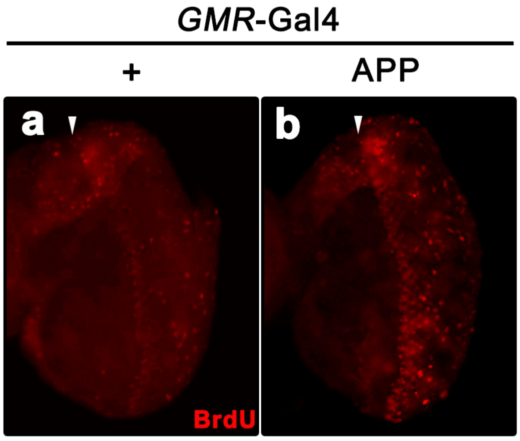

Supplement: Supplementary Information [file srep16816-s1.pdf]
